# Improving Overall Survival and Quality of Life in Patients with Prostate Cancer and Neuroendocrine Tumors Using ^177^Lu-iPSMA and ^177^Lu-DOTATOC: Experience after 905 Treatment Doses

**DOI:** 10.3390/pharmaceutics15071988

**Published:** 2023-07-20

**Authors:** Myrna Luna-Gutiérrez, Rodrigo Hernández-Ramírez, Airam Soto-Abundiz, Osvaldo García-Pérez, Alejandra Ancira-Cortez, Sergio López-Buenrostro, Brenda Gibbens-Bandala, Irma Soldevilla-Gallardo, Nancy Lara-Almazán, Melissa Rojas-Pérez, Blanca Ocampo-García, Erika Azorín-Vega, Clara Santos-Cuevas, Guillermina Ferro-Flores

**Affiliations:** 1 Department of Radioactive Materials, Instituto Nacional de Investigaciones Nucleares (ININ), Ocoyoacac 52750, Mexicoerica.azorin@inin.gob.mx (E.A.-V.); clara.cuevas@inin.gob.mx (C.S.-C.); 2 Department of Nuclear Medicine, Hospital Médica Sur, Mexico City 14080, Mexico; 3 Department of Nuclear Medicine, Instituto Nacional de Cancerología, Mexico City 14000, Mexico; 4 Imaging Division, Unidad de Patología Clínica e Imagenología, Guadalajara 44650, Mexico; 5 Department of Nuclear Medicine, Centro Médico ABC Campus Observatorio, Mexico City 01120, Mexico

**Keywords:** PSMA, somatostatin receptors, lutetium-177, ^177^Lu-DOTATOC, ^177^Lu-iPSMA

## Abstract

^177^Lu-iPSMA is a novel radioligand developed at ININ-Mexico with a high affinity for the PSMA protein heavily expressed in cancer cells of approximately 95% of patients with metastatic castration-resistant prostate cancer (mCRPC). ^177^Lu-DOTATOC is a patent-free radioligand, molecularly recognized by somatostatin receptors (SSTR-2) overexpressed in cancer cells of about 80% of patients with metastatic gastroenteropancreatic neuroendocrine tumors (GEP-NET). This translational research aimed to determine the efficacy and safety of ^177^Lu-iPSMA and ^177^Lu-DOTATOC developed as GMP pharmaceutical formulations for treating progressive and advanced mCRPC and NET. One hundred and forty-five patients with mCRPC and one hundred and eighty-seven subjects with progressive NET (83% GEP-NET and 17% other NET), treated with ^177^Lu-iPSMA and ^177^Lu-DOTATOC, respectively, were evaluated. Patients received a mean dose of 7.4 GBq per administration of ^177^Lu-iPSMA (range 1–5 administrations; 394 treatment doses) or ^177^Lu-DOTATOC (range 2–8 administrations; 511 treatment doses) at intervals of 1.5–2.5 months. Efficacy was assessed by SPECT/CT or PET/CT. Results were stratified by primary tumor origin and number of doses administered. Patients with mCRPC showed overall survival (OS) of 21.7 months with decreased radiotracer tumor uptake (SUV) and PSA level in 80% and 73% of patients, respectively. In addition, a significant reduction in pain (numerical scale from 10–7 to 3–1) was observed in 88% of patients with bone metastases between one and two weeks after the second injection. In the GEP-NET population, the median progression-free survival was 34.7 months, with an OS of >44.2 months. The treatments were well tolerated. Only ten patients experienced grade ≥ 3 myelosuppression (3% of all patients). The observed safety profiles and favorable therapeutic responses demonstrated the potential of ^177^Lu-iPSMA and ^177^Lu-DOTATOC to improve overall survival and quality of life in patients with progressive and advanced mCRPC and NET.

## 1. Introduction

Metastatic castration-resistant prostate cancer (mCRPC) is a fatal disease that follows an aggressive course with patients dying rapidly [1]. In general, androgen receptor pathway inhibitor (ARPI) hormone therapy (e.g., abiraterone, enzalutamide, and apalutamide) and different taxanes-based chemotherapy regimens (cabazitaxel, docetaxel, and paclitaxel) achieve minimal improvement in these patients, resulting in progression of the disease [2]. The prostate-specific membrane antigen (PSMA) is heavily expressed in 95% of cancer cells in patients with mCRPC. The expression degrees of PSMA directly associate with metastasis, progression, and androgen independence in prostate cancer [3]. Lutetium-177 is a radionuclide that emits β radiation particles with a maximum tissue penetration of 2.2 mm (mean penetration 0.67 mm; maximum energy of 497 keV), sufficient to kill target tumor cells with a limited effect on neighboring normal cells. Thus, PSMA is used as a molecular target for radiotherapy of advanced mCRPC using specific ^177^Lu-radiopharmaceuticals as a third or fourth line of treatment, improving overall survival and analgesic control in the final phase of the disease [4,5].

Somatostatin receptors are commonly overexpressed in progressive and advanced neuroendocrine tumors (NET) [6]. Twenty percent of the patients with NET present distant metastases, cases in which tumors are no longer resectable. Treatment options for unresectable metastatic disease include octreotide therapy (somatostatin analogs), first-line therapy, and targeted radiotherapy or interferon-alpha as second-line therapies [7].

Pain is one of the most complicated symptoms in cancer patients, which negatively affects quality of life, especially in patients with prostate cancer bone metastases. Even with all the current scientific advances, the only option medical oncology can offer patients with advanced cancer is prolonged survival and improved quality of life (including pain relief), two aspects in which targeted radiotherapies with lutetium-177 have proven highly effective [7,8]. Unfortunately, only ^177^Lu-PSMA-617 (Pluvicto^TM^, Novartis, Millburn, NJ, USA) and ^177^Lu-DOTATATE (Lutathera^TM^, Novartis, Zaragoza, Spain) have been approved by the FDA (US Food and Drug Administration) as patented products for the treatment of advanced mCRPC and NET, respectively. Therefore, given the need for targeted radiotherapies to benefit a greater significant number of cancer patients whose cases are growing at an accelerated rate, we need to develop our own technology to enhance the benefits of lutetium-177 radiopharmaceuticals. Recently, we reported the design, synthesis, pharmaceutical formulation, preclinical evaluation, and assessment of biokinetics and dosimetry in patients of a novel ^177^Lu-labeled PSMA inhibitor (^177^Lu-iPSMA) (Mexico-ININ) for prostate cancer therapy [9,10,11], as well as the pharmaceutical grade preparation of ^177^Lu-DOTATOC as a patent-free radioligand molecularly recognized by somatostatin receptors (SSTR-2) for NET treatment [11].

This translational research aimed to determine the efficacy and safety of ^177^Lu-iPSMA and ^177^Lu-DOTATOC developed as GMP pharmaceutical formulations for the treatment of progressive and advanced mCRPC and NET.

## 2. Materials and Methods

### 2.1. Preparation of Radiotracers

^177^LuCl_3_ (40 GBq/mL, non-carrier-added in sterile 0.04 M HCl solution) was purchased from ITM, Garching bei München, Germany (EndolucinBeta). Analytical grade reagents were obtained from Merck Co., Boston, MA, USA and were used as received. ^177^Lu-iPSMA and ^177^Lu-DOTATOC were prepared according to the procedure reported by Luna-Gutierrez et al. [11] using sterile and apyrogenic multidose lyophilized kits (ININ-Mexico) under GMP conditions. In brief, 1.5 mL of 1 M acetate buffer pH 5.0 was added to the ^177^LuCl_3_ vial (40 GBq/mL). The total volume was used for the reconstitution of the iPSMA (DOTA-hydrazinylnicotinoyl-lysine(Nal)-urea-glutamate) or DOTATOC (DOTA-Tyr^3^-octreotide) lyophilized kits followed by heating at 95 °C for 30 min (in a dry bath). Finally, the volume was adjusted to 20 mL with injectable-grade water (Pisa, Mexico). Dosing was performed in syringes using a GMP module (Musa, Comecer, Castel Bolognese, Italy) (7.4 GBq/4 mL). Under this procedure, ^177^Lu-iPSMA was prepared from lyophilized kits using sterile solutions of ^177^LuCl_3_ for kit reconstitution without further purification or sterilization steps and radiochemical synthesizers. The quality control procedure evaluated parameters such as radiochemical purity (reversed-phase HPLC), sterility, bacterial endotoxins, pH, color, and appearance using the Mexican Pharmacopeia methods. 

^68^Ga was obtained from a ^68^Ge/^68^Ga generator (ITM, Baden-Württemberg, Germany). ^68^Ga-iPSMA and ^68^Ga-DOTATOC were synthesized on an iQS Ga-68 Fluidic Labeling Module (ITG, Baden-Württemberg, Germany). [^99m^Tc]TcO_4_Na was obtained from a GETEC (^99^Mo/^99m^Tc generator) (ININ, La Marquesa, Mexico). [^99m^Tc]Tc-iPSMA and [^99m^Tc]Tc-Tyr^3^-octreotide radiotracers were prepared from lyophilized kits (ININ-Mexico), as previously reported [12,13].

### 2.2. Radiochemical Purity Assessment

Radiochemical purities over 95% were verified by radio-HPLC (Waters; Empower Software; Orlando, FL, USA) using a C18 column (µBondapak: 10 µm, 3.9 × 300 mm). A linear gradient in 20 min from 100% to 25% of solvent A (0.1% TFA/water) and from 0% to 75% of solvent B (0.1% TFA/acetonitrile) at a 1 mL/min flow rate was used. Retention times were as follows: 3–4 min (^177^LuCl_3_, [^99m^Tc]TcO_4_Na, and ^68^GaCl_3_), 14–15 min (^177^Lu-iPSMA and ^177^Lu-DOTATOC), 13–14 min ([^99m^Tc]Tc-iPSMA and [^99m^Tc]Tc-Tyr^3^-octreotide), and 10–12 min (^68^Ga-iPSMA and ^68^Ga-DOTATOC).

### 2.3. Patients Characteristics and General Protocols

The ^177^Lu-iPSMA study enrolled 145 patients (median age: 69 years; range 57–86 years) with histologically-confirmed prostate cancer from four Mexican hospital institutions. Patients presented recurrent disease and confirmed biochemical progression (3 consecutive PSA elevations, separated by at least one week with PSA > 40 ng/mL) or radiological progression (2 or more bone lesions or in soft tissue or lymph node lesions). The Gleason score was 8 to 10 in 87% of the cases, and the rest of the patients had no score report. Most of the patients had previously been treated with Abiraterone or Enzalutamide (androgen receptor pathway inhibitor) with or without systemic chemotherapy (taxanes therapy) or radiation therapy (Table 1). The primary diagnosis of prostate cancer was established in 2007–2019. 

Patients received from 1 to 5 doses of ^177^Lu-iPSMA (7.4 GBq/dose, 120 µg of iPSMA peptide) every six weeks between January 2016 and May 2023. The number of ^177^Lu-iPSMA doses to be administered in each treatment was determined based on the tumor volume and the tumor standardized uptake value (SUVmax), estimated by ^99m^Tc-iPSMA SPECT/CT or ^68^Ga-iPSMA PET/CT (radiological molecular imaging) [10]. For example, tumor burden is related to the number of organic systems involved, including previous hematopoietic damage [14]. Therefore, patients with higher bone tumor burden were selected to receive a maximum of 3 to 4 doses of ^177^Lu-iPSMA due to the increased likelihood of myelotoxicity. The biochemical and clinical condition of the patients was the second criterion for determining the number of doses to be administered. For the evaluation of survival, only patients who completed the administration of 3 to 5 doses of ^177^Lu-iPSMA between January 2016 and January 2020 were included (*n* = 52). Time and cause of death were also recorded. Patients who died from causes other than prostate cancer were followed for cancer-specific survival until censoring. This group was compared with a historical control group of patients with mCRPC (*n* = 31), which was retrospectively analyzed, given that they were diagnosed in the period 1989–2003, prior to the routine use of docetaxel, and were treated exclusively with antiandrogen withdrawal and palliative measures, which generally included analgesic management and oral prednisone (10 mg) in the terminal phase of the disease, as previously reported [15]. Kaplan–Meier analysis, log-rank test, and proportional hazards analysis were performed. The response was evaluated using [^99m^Tc]Tc-iPSMA-SPECT/CT or ^68^Ga-iPSMA PET/CT images and serum PSA levels before and after ^177^Lu-iPSMA treatment. Patients initially selected with ^99m^Tc-iPSMA/SPECT quantitative imaging were followed with the same imaging modality throughout the study, as were patients followed with ^68^Ga-iPSMA/PET. Radiologic progression-free survival was also determined from the start of targeted radiotherapy until disease progression. 

The ^177^Lu-DOTATOC study enrolled 187 patients (83 men, median age: 64 years; range 51–81 years; and 104 women, median age: 63 years; range 48–74 years) diagnosed with metastatic and progressive NET from four Mexican hospital institutions. Eighty-three percent of the cases (*n* = 155) were patients with gastroenteropancreatic neuroendocrine tumors (GEP-NET: 67% gastroenteric and 33% pancreatic NET) and 17% (*n* = 32) other NETs (9 kidney, 17 lung, 5 ovary, and 1 thymus). Ninety-eight percent of the patients were previously treated with long-acting somatostatin analogs.

Patients received 2 to 8 doses of ^177^Lu-DOTATOC (7.4 GBq/dose, 200 µg of DOTATOC peptide) with co-infusion of an amino acid solution (hydrochloride L-lysine-25 g/hydrochloride L-arginine-25 g/0.9% NaCl) every 8 to 10 weeks between January 2016 and May 2023. For the evaluation of survival, only patients with NET who completed the administration of 4 to 8 doses of ^177^Lu-DOTATOC between January 2016 and January 2019 were included (*n* = 81). The time and cause of death were recorded. Patients who died from causes other than NET provided cancer-specific survival follow up until they were censored. Kaplan–Meier analysis was carried out. The response was evaluated using CT and [^99m^Tc]Tc-Tyr^3^-octreotide-SPECT/CT or ^68^Ga-DOTATOC-PET/CT, and the clinical and biochemical (chromogranin A and serotonin levels) patient condition before and after therapy. 

After ^177^Lu-iPSMA or ^177^Lu-DOTATOC administration, patients were hydrated with 0.5 L of water with urination before the radiological/nuclear image acquisition. All patients underwent [^99m^Tc]Tc-MAG3 renal scintigraphy. Additional blood counts and laboratory parameters were performed to rule out clinically relevant bone marrow depression, hepatic function, and renal impairment. Each patient signed written informed consent.

The Hospital Institutional Review Boards approved the clinical trials for lutetium-177 therapies in compliance with the following approvals: (a) COFEPRIS (Federal Commission for Protection against Health Risks, the regulatory authority in Mexico) approval for ^177^Lu-iPSMA use in patients with progressive and advanced prostate cancer and ^177^Lu-DOTATOC use in patients with progressive and advanced NET (Registration numbers: 2763R2017 SSA, 2674R2016 SSA, and 1115R2019 SSA), under the ethical standards of the responsible committee on human studies (institutional and national) and with the Helsinki Declaration of 1975, as revised in 2008; (b) the GMP certificate issued by COFEPRIS to ININ facilities for the preparation of radiopharmaceuticals; and (c) the clinical background of PSMA inhibitors and Tyr^3^-octreotide for imaging approved by COFEPRIS (Registration numbers: 2764R2017 SSA and 0502R2017 SSA, respectively). The clinical protocol adhered to the ethical codes of objectivity, professional conduct, integrity, professional competence and due care, and confidentiality. Patients were informed that their identity and medical information would not be disclosed at any time. Therefore, in the collection of clinical data, patients would not face any risks related to the protection of confidentiality, which would be protected by coding his/her samples and information. The code is an identification number that does not contain personal information. Patients were also informed that their participation was voluntary. If the patient decided not to participate, it would not affect his/her relationship with his/her health care institution or his/her right to receive medical care or services to which he/she was entitled. If the patient decided to participate, he/she was free to withdraw consent and discontinue participation at any time without affecting his/her care by the health care institution.

## 3. Results

### 3.1. ^177^Lu-iPSMA

**Figure 1:**
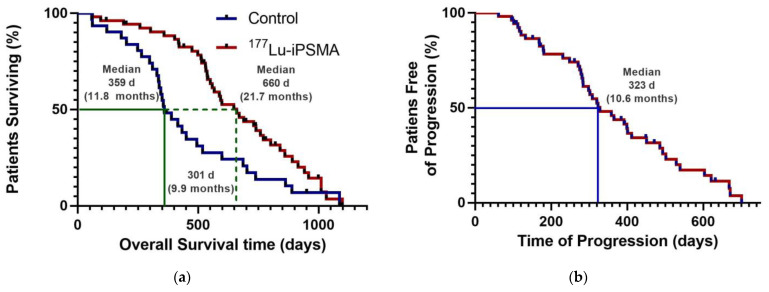
Kaplan–Meier plots: (**a**) Overall survival for patients with progressive metastatic castration-resistant prostate cancer (mCRPC) treated with three to five doses of ^177^Lu-iPSMA (7.4 GBq/dose). Stratification in the group treated with ^177^Lu-iPSMA (red) and control group (blue). (**b**) Radiological progression-free survival for patients treated with three to five doses of ^177^Lu-iPSMA (7.4 GBq/dose).

Patients with mPC who received three to five doses of ^177^Lu-iPSMA showed an overall survival of 660 days (21.7 months) (Table 2; Figure 1a) with decreased radiotracer tumor uptake (SUV) and PSA level in 80% (*n* = 33) and 73% (*n* = 30) of the subjects, respectively (Table 2). Specifically, 52% of cases had a PSA decline of >50% after completing treatment. 

The hazard ratio was 0.5650 (95% CI: 0.3391–0.9412), indicating a 43.5% reduction in the risk of death in favor of the ^177^Lu-iPSMA group (Table 2). In addition, 50% of patients were free of progression at 323 d (10.6 months) after treatment (Figure 1b).

**Figure 2:**
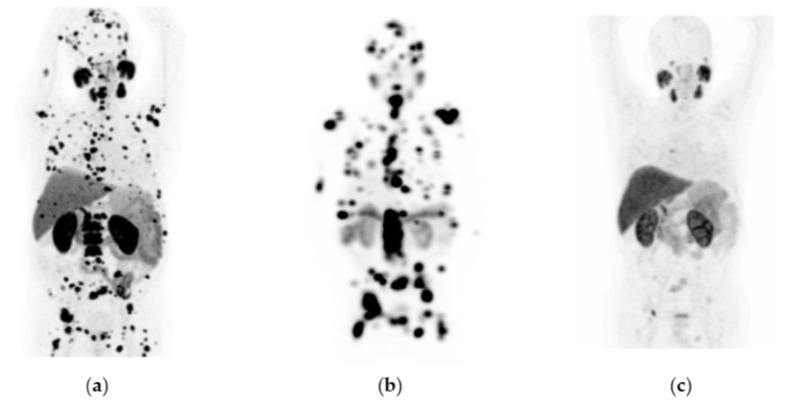
Patient with progressive and metastatic castration-resistant prostate cancer (mCRPC): (**a**) ^68^Ga-iPSMA PET/CT imaging before ^177^Lu-iPSMA therapy; initial levels of hemoglobin (15.5 g/dL), leucocytes (5700/µL), creatinine (0.82 mg/dL), platelets (238,000/µL), and serum PSA (129 ng/mL). (**b**) ^177^Lu-iPSMA SPECT imaging (first dose). (**c**) ^68^Ga-iPSMA PET/CT imaging eight months after the first ^177^Lu-iPSMA dose was administered; levels of hemoglobin (13.4 g/dL), leukocytes (3900/µL), creatinine (0.79 mg/dL), platelets (277,000/µL), and serum PSA (1.5 ng/mL). The patient responded completely to ^177^Lu-iPSMA therapy (four doses every six weeks; 7.4 GBq/dose).

**Figure 3:**
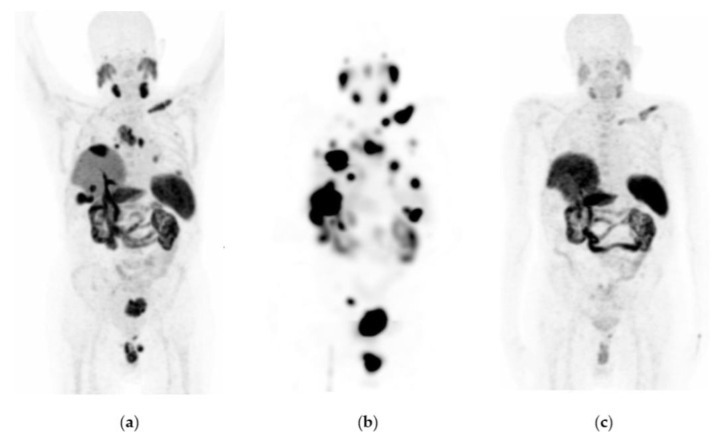
Patient with progressive and metastatic castration-resistant prostate cancer (mCRPC): (**a**) ^68^Ga-iPSMA PET/CT imaging before ^177^Lu-iPSMA therapy; initial levels of hemoglobin (11.1 g/dL), leucocytes (4900/µL), creatinine (0.88 mg/dL), platelets (115,000/µL), and serum PSA (84.7 ng/mL). (**b**) ^177^Lu-iPSMA SPECT imaging (first dose). (**c**) ^68^Ga-iPSMA PET/CT imaging 10 months after the first ^177^Lu-iPSMA dose was administered; levels of hemoglobin (10.1 g/dL), leukocytes (2100/µL), creatinine (0.91 mg/dL), platelets (87,000/µL), and serum PSA (7.7 ng/mL). The patient responded partially to ^177^Lu-iPSMA therapy (four doses every six weeks; 7.4 GBq/dose).

**Figure A1:**
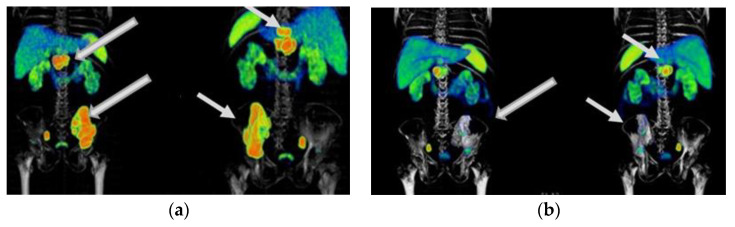
Patient with mCRPC who had a partial response to ^177^Lu-iPSMA treatment: (**a**) anterior and posterior ^68^Ga-iPSMA imaging before ^177^Lu-iPSMA treatment, and (**b**) after ^177^Lu-iPSMA therapy (four doses of 7.4 GBq). Arrows indicate tumor lesion sites.

**Figure A2:**
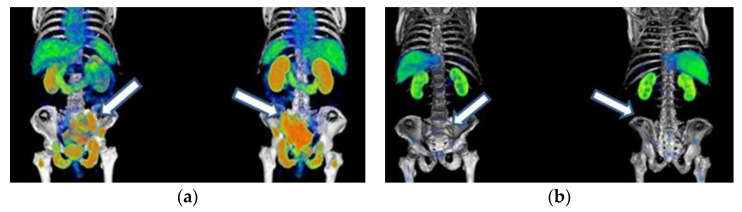
Patient with mCRPC who had a complete response to ^177^Lu-iPSMA treatment: (**a**) anterior and posterior ^68^Ga-iPSMA imaging before ^177^Lu-iPSMA treatment, and (**b**) after ^177^Lu-iPSMA therapy (three doses of 7.4 GBq). Arrows indicate tumor lesion sites.

**Figure A3:**
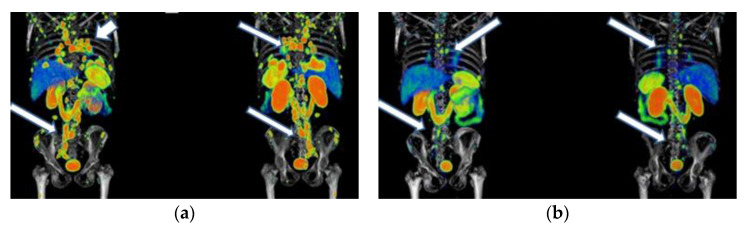
Patient with mCRPC who had a partial response to ^177^Lu-iPSMA treatment: (**a**) anterior and posterior ^68^Ga-iPSMA imaging before ^177^Lu-iPSMA treatment, and (**b**) after ^177^Lu-iPSMA therapy (three doses of 7.4 GBq). Arrows indicate tumor lesion sites.

^177^Lu-iPSMA showed an overall response rate of 36.6% (Table 2). Figure 2 and Figure 3 show typical clinical cases of complete response (disappearance of all lesions or adenopathies less than 10 mm short axis) and partial response (decrease of at least 30% in the sum of the largest diameters of lesions compared with the baseline study) after ^177^Lu-iPSMA therapy. In addition, the reduction of large tumors and multiple metastatic lesions are proof of the high specificity and molecular recognition of ^177^Lu iPSMA by PSMA at the tumor cell level (Appendix A) (Figure A1, Figure A2 and Figure A3).

In the group of patients who received one to two doses of ^177^Lu-iPSMA (7.4 GBq/dose) in the period from January 2016 to January 2020 (*n* = 26), a high percentage of censored cases was registered (69%; *n* = 18), and the rest of them (*n* = 8) died from progressive disease prior to further ^177^Lu-iPSMA radiotherapy (progressive disease: at least a 20% increase in the sum of tumor lesion diameters, using the smallest sum in the study as baseline).

Of the rest of the patients who have received from one to five doses between February 2020 and May 2023 (*n* = 67), 69 percent (*n* = 46) have not yet completed their treatment or remain alive with stable disease (free of progression); 13 percent (*n* = 9) were censored cases, and 18 percent (*n* = 12) died because of disease progression. However, 78% of this group of patients (*n* = 52) had a Karnofsky score between 90 and 70 after administering the second treatment dose. Furthermore, of patients with bone metastases (*n* = 109), 83% (*n* = 90) showed a significant decrease in pain (numerical scale from 10–7 to 3–1), which undoubtedly improved their quality of life.

In 40% of treated patients, adverse reactions were dry mouth, fatigue, and nausea, and less frequently (15% of patients), decreased appetite and constipation. These negative effects usually disappeared two months after administering the last dose of ^177^Lu-iPSMA. However, 19% of subjects treated with three to five doses developed permanent xerostomia. The decrease in hemoglobin, leukocytes, and platelets in 60% of the treated cases (myelosuppression grade 1 to 2) returned to baseline three months after concluding the treatment. Only three patients (6%) presented myelosuppression grade 3. There was no evidence of hepatotoxicity or nephrotoxicity.

### 3.2. ^177^Lu-DOTATOC

The results were classified according to the origin of the primary tumors [group 1: GEP-NT (*n* = 53), and group 2: other NTs (*n* = 28)] and the number of doses administered [group 3: GEP-NT three to four doses (*n* = 39); and group 4: GEP-NT five to six doses (*n* = 14)]. The other NET group received three to four doses (*n* = 28). The median follow up was 36 months (36 ± 16 months) for patients included at the January 2019 cut-off date (*n* = 81) in each study group.

**Figure 4:**
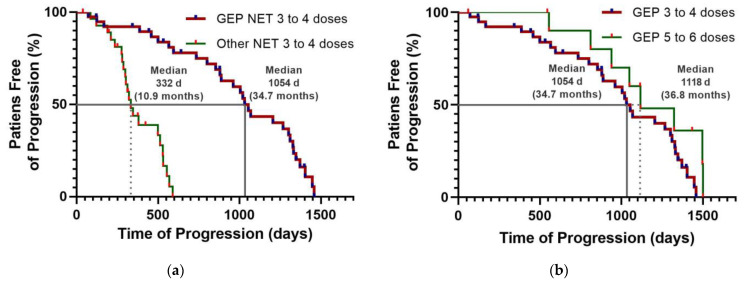
Kaplan–Meier plots: (**a**) Progression-free survival for patients with metastatic neuroendocrine tumors treated with ^177^Lu-DOTATOC (7.4 GBq/dose). Stratification for GEP-NET (red) and other-NET (green) groups. (**b**) Progression-free survival for patients treated with three to four and five to six doses of ^177^Lu-DOTATOC (7.4 GBq/dose).

Fifty percent of patients with GEP-NET who received three to five doses of ^177^Lu-DOTATOC were free of progression at 1054 d (34.7 months) (Table 3; Figure 4a); while in the NET group (other NET different from GEP-NET), it was 332 days (10.9 months) (Table 3; Figure 4a). The hazard ratio was 0.1742 (95% CI: 0.05854–0.5182), indicating an 82.6% reduction in the risk of disease progression in favor of the GEP-NET group treated with ^177^Lu-DOTATOC (Table 3). Fifty percent of patients in the GEP-NET group who received three to four doses of ^177^Lu-DOTATOC were free from progression at 34.7 months, compared to 36.8 months in the group who received five to six doses (Figure 4b). However, the survival curves were not significantly different, with a hazard ratio of 0.5402 (95% CI: 0.275–1.062; *p* = 0.2260, unstratified bilateral log-rank test) in favor of the GEP-NET group receiving five to six doses.

**Figure 5:**
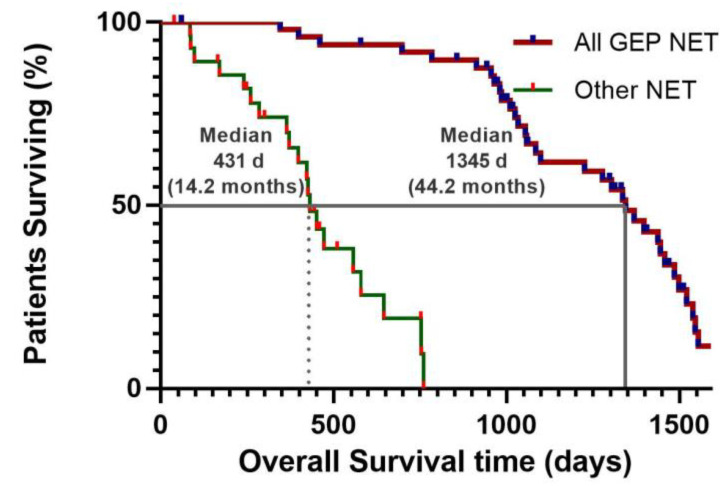
Kaplan–Meier plots: Overall survival for patients with neuroendocrine tumors (GEP-NET and other NET) treated with three to six doses of ^177^Lu-DOTATOC (7.4 GBq/dose).

Follow up was 52 months at the time of the OS analysis, which was performed after the randomization of the last patient. Median OS was prolonged to a clinically relevant extent of 1345 day (44.2 months) in patients of the GEP-NET group compared with patients in the other-NET group with an OS of 431 days (14.2 months) (Table 3) (Figure 5). However, at the end of the study, the median overall survival of patients with GEP-NET treated with ^177^Lu-DOTATOC had not been reached, as three patients, two on the six-dose regimen and one on the four-dose regimen, were alive. Therefore, OS is >44.2 months (Figure 5).

An overall response rate of 52% was observed in patients with GEP-NT treated with ^177^Lu-DOTATOC (Table 3). As expected, most cases were partial responses (47%) and stable disease (45%), with a disease control rate of 97%. No patient had a complete or partial response in the group of patients with other NETs. However, they had a disease control rate of 71% (Table 3).

**Figure 6:**
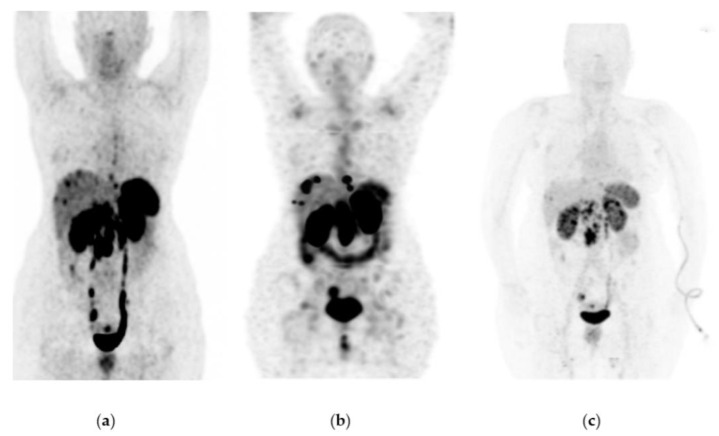
A 65-year-old woman was diagnosed with a well-differentiated neuroendocrine tumor of the intestine with metastases to the liver, mesenteric nodes, and abdominal implants. Due to disease progression, she was considered a candidate for therapy with ^177^Lu-DOTATOC (four doses of 7.4 GBq) in combination with octreotide. Response: partial response. She did not show any hematologic side effects. The last renal scintigraphy showed a slight decrease in renal tubular function with no changes compared to previous scintigraphy. (**a**) ^68^Ga-DOTATOC PET/CT imaging before ^177^Lu-DOTATOC therapy, (**b**) ^177^Lu-DOTATOC SPECT imaging (first dose), (**c**) ^68^Ga-DOTATOC PET/CT imaging one year after the first ^177^Lu-DOTATOC therapy.

**Figure 7:**
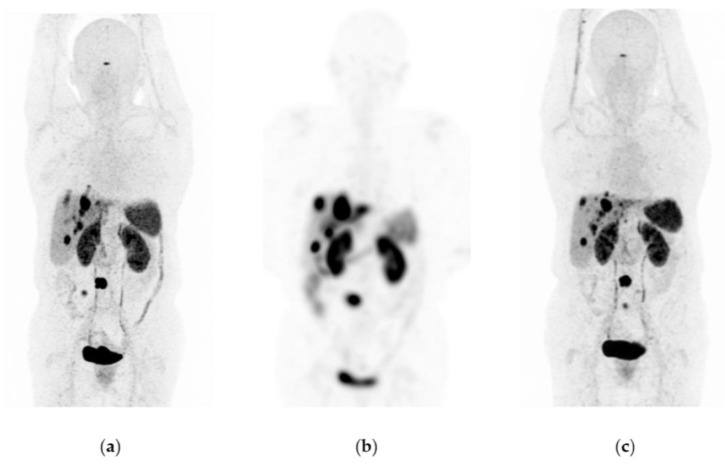
A 48-year-old woman was diagnosed with a metastatic neuroendocrine tumor of the ileum. She received ^177^Lu-DOTATOC (four doses of 7.4 GBq) in combination with octreotide. Response: stable disease. No hematologic or renal toxicity. (**a**) ^68^Ga-DOTATOC PET/CT imaging before ^177^Lu-DOTATOC therapy, (**b**) ^177^Lu-DOTATOC SPECT imaging (first dose), (**c**) ^68^Ga-DOTATOC PET/CT imaging one year after the first dose of ^177^Lu-DOTATOC.

**Figure 8:**
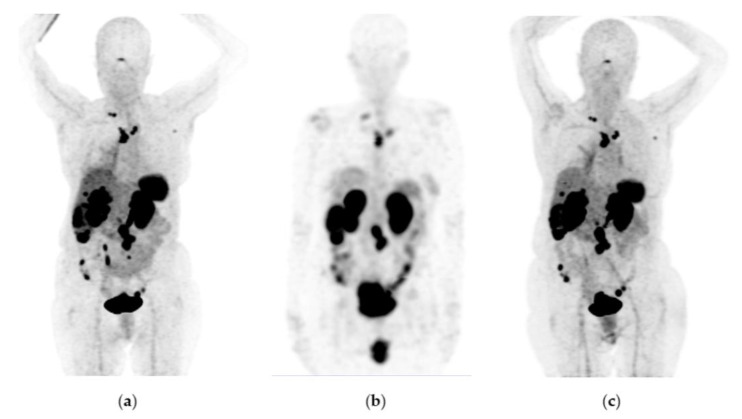
A 76-year-old female patient received four doses of ^177^Lu-DOTATOC with stable disease as determined by ^68^Ga-DOTATOC PET/CT imaging. Findings: 11% overall increase in somatostatin receptor expression in the primary pancreatic tumor lesion, as well as metastatic deposits in lymph nodes, liver, and peritoneal implants toward the root of the mesentery, jejunum, and pelvic cavity, without significant morphologic changes. Data refer to a stable disease. (**a**) ^68^Ga-DOTATOC PET/CT imaging before ^177^Lu-DOTATOC therapy, (**b**) ^177^Lu-DOTATOC SPECT imaging (first dose), (**c**) ^68^Ga-DOTATOC PET/CT imaging ten months after the first dose of ^177^Lu-DOTATOC.

Figure 6, Figure 7 and Figure 8 show typical clinical cases of partial response and stable disease after ^177^Lu-DOTATOC therapy (the stable disease does not meet the criteria for partial response or progressive disease).

Of the rest of the patients who received from two to eight doses between February 2019 and May 2023 (*n* = 105), 58 percent (*n* = 61) have not yet completed their treatment or remain alive with stable disease; 22 percent (*n* = 23) were censored cases, and 20 percent (*n* = 21) died because of disease progression. However, 81% of patients had a Karnofsky score over 80 after administering the second treatment dose, significantly improving their quality of life. 

In 48% of treated patients, adverse reactions were nausea and vomiting during perfusion of the radiopharmaceutical and co-administration of amino acids. Less frequently (19.5%), fatigue and decreased appetite were also presented. The decrease in hemoglobin, leukocytes, and platelets in 23.2% of the treated cases (myelosuppression grade 1 to 2) returned to baseline three months after concluding the treatment. Grade ≥ 3 of neutropenia, thrombocytopenia, or lymphopenia occurred in 4% (*n* = 7) of the total patients (*n* = 187) treated with ^177^Lu-DOTATOC. There was no evidence of hepatotoxicity or significant correlation between the assessment of renal function (biochemistry and dynamic nuclear imaging for renal function testing) and the use of therapeutic doses of ^177^Lu-DOTATOC that could indicate any moderate or severe nephrotoxicity.

## 4. Discussion

The clinical results of this translational study are consistent with the dozens of published clinical studies on the efficacy and toxicity of various ^177^Lu-labeled ligands for the treatment of advanced and progressive mCRPC and GEP-NET [7,8,16,17,18].

It is important to note that, in line with the clinical results of these clinical trials, we have demonstrated that a maximum of three to five doses of ^177^Lu-iPSMA and three to four doses of ^177^Lu-DOTATOC are sufficient to achieve highly favorable response rates in terms of quality of life, with a low probability of serious side effects such as myelotoxicity (grade ≥ 3) or renal damage, which, far from being beneficial to patients, further compromise their critical health status.

^177^Lu-iPSMA therapy is a convenient option for the treatment of multiple metastases and large tumor lesions (Appendix A) (Figure A1, Figure A2 and Figure A3). For example, in the case shown in Figure A1, the patient suffered from severe pain characteristic of the terminal stage of the disease. However, two weeks after administration of the first dose of ^177^Lu-iPSMA, the subject showed relevant pain relief. Furthermore, after administering four doses of ^177^Lu-iPSMA (four doses of 7.4 GBq), metastatic lesions in the hip and spine were significantly reduced, prolonging the patient’s life for 19 months after therapy. In this context and based on the results of this study, ^177^Lu-iPSMA therapy should be considered and evaluated in future clinical trials as a second-line rather than third- or fourth-line therapy in patients with mCRPC, with the potential to improve patient survival.

For more than a decade, peptide-targeted radionuclide therapies using lutetium-177 have experienced remarkable growth worldwide as they have demonstrated their ability to improve overall survival and quality of life in patients with advanced cancer. This fact has opened an important line of research for the development of new molecular sensors capable of delivering the radionuclide Lu-177 to tumor sites in a highly specific manner. 

In prostate cancer and neuroendocrine tumors, ^177^Lu-PSMA-617 and ^177^Lu-DOTATATE are the radiopharmaceuticals with the most reported phase III clinical trials. ^177^Lu-iPSMA, whose clinical cases are reported in this research, is a new peptide ligand containing a pyridine ring and a hydrazine group that provides an additional anchoring site to target the PSMA enzyme and a molecular spatial configuration that favors its ability to remain in tumor lesions to produce ablative doses of radiation in a highly selective mode [9,10]. Therefore, this is the first report of a clinical trial of ^177^Lu-iPSMA in a significant number of patients with mCRPC. Our results showed an overall survival (OS) of 21.7 months. This finding is relevant and comparable to ^177^Lu-PSMA-617, for which an OS of 15.3 months is reported [19,20]. Of course, to determine whether there is a real difference between ^177^Lu-iPSMA and ^177^Lu-PSMA-617, a specific clinical trial would have to be performed under the same conditions and clinical parameters. Nevertheless, there is clearly a need for a greater number of new clinically proven ligands, such as iPSMA, which can be labeled with therapeutic radionuclides to treat these patients, given the 2020 data for prostate cancer, where 375,000 deaths and 1.4 million new cases were diagnosed in one year (the most diagnosed cancer in 112 countries) [21].

On the other hand, ^177^Lu-DOTATOC is commonly prepared using commercially available radiochemical synthesizers connected to ISO Class 5 areas. However, the radiochemical yield using synthesizers ranges from 74 to 90% [11]. Previously, we reported the use of multi-dose kit formulations to produce ^177^Lu-DOTATOC with radiochemical yields greater than 95% in a rapid process requiring only reconstitution of a lyophilized vial followed by incubation in a dry bath for 30 min [11]. Therefore, this is the first time that clinical results in a significant number of patients have been reported for ^177^Lu-DOTATOC prepared from lyophilized formulations manufactured under GMP conditions. Considering that clinical reports for ^177^Lu-DOTATOC are scarce compared to ^177^Lu-DOTATATE (only one Phase II and no Phase III clinical trials have been reported for ^177^Lu-DOTATOC [17]), the results reported in this research provide relevant clinical data for the consolidation of ^177^Lu-DOTATOC by demonstrating that it shows as high efficacy as ^177^Lu-DOTATATE in the treatment of patients with advanced neuroendocrine tumors. It is important to note that the clinical results of most clinical publications of ^177^Lu-DOTATOC and ^177^Lu-DOTATATE generally include patients with advanced and unresectable NET as inclusion and overall outcome characteristics. In this study, it was clearly demonstrated that in the GEP-NET population, the median progression-free survival was 34.7 months with an OS of >44.2 months, while the benefit in the other types of NET is only an OS of 14.2 months. This finding is consistent with the only reported phase II study of ^177^Lu-DOTATOC [17] and provides a strong rationale for further phase III clinical trials specifically targeting GEP-NETs.

Finally, the retrospective nature of the control group study for ^177^Lu-iPSMA and the still limited sample size in both the ^177^Lu-iPSMA and ^177^Lu-DOTATOC trials are the main limitations of the present study. Nonetheless, the results obtained are promising and warrant further prospective phase III trials of PRRT in patients with mCRPC and NET to confirm or refute our findings.

Severe pain, which increases with disease progression, affects approximately 64% of patients with mCRPC or NET tumors. This condition has a direct impact on survival and quality of life in ways that are dependent on proper pain management. The challenge is to find a balance between pain control and analgesia so that the patient remains independent enough to perform daily activities [22].

In current clinical practice, adequate pain control is achieved when the focus of palliative treatment is on targeted radiation therapy [23]. It is important to note that cancer pain is thought to result from local inflammation due to mechanical compression of nerve terminals and tumor surrounding tissues [24]. However, recent evidence suggests that the main mechanisms of cancer pain are the loss of homeostasis, anatomical remodeling, and neurochemical changes that occur between tumor, neuronal, immune, and stromal cells [24]. These dynamic intercellular changes include the secretion of prostaglandins, interleukins, and growth factors that promote acidification of the environment, which remodels the relationship between osteoclasts and osteoblasts and alters the ion channel [25]. In addition, the density of neuropathic and nociceptive receptors triggers neurochemical changes such as sensitization, with the consequent opening and closing of ion channels, altering the endogenous electrochemical potential of nerve fibers innervating bone [25,26]. Lutetium-177 radiopharmaceuticals are a fast and effective palliative treatment option. Unlike chemotherapy, they do not cause neuropathic pain as an adverse effect [27]. As shown in this translational clinical trial, ^177^Lu-iPSMA and ^177^Lu-DOTATOC significantly reduce cancer pain. Thus, modern radiobiology suggests that the mechanism that drives pain relief is the reduction on ionic flux through the functional expression of voltage-gated ion channels, generating a radiation-induced nerve rearrangement [28].

## 5. Conclusions

The current study supports the safety and efficacy findings of prolonged progression-free and overall survival, improved quality of life, and low toxicities in patients treated with three to five doses of ^177^Lu-iPSMA (7.4 GBq/dose). In addition, the observed safety profiles and favorable therapeutic responses demonstrated the potential of ^177^Lu DOTATOC (three to four doses; 7.4 GBq/dose) to improve the quality of life in patients with progressive and advanced neuroendocrine tumors, particularly gastroenteropancreatic tumors.

## 6. Patents

^177^Lu-DOTA-HYNIC-iPSMA as a therapeutic radiopharmaceutical targeting prostate-specific membrane antigen. Mexican Patent No. 380340. European Patent No. 3766893

## Figures and Tables

**Table 1:** Patient characteristics before ^177^Lu-iPSMA treatments.

	Patients (*n* = 145)	
Site of Metastases	*n*	%
Bone	109	75
Lymph nodes	138	95
Liver	61	42
Lung	3	2
**Prior therapies**		
Radical prostatectomy	91	63
Radiation therapy (prostate region)	120	83
Cabazitaxel, Docetaxel (taxanes therapy)	131	90
Abiraterone and/or Enzalutamide (androgen receptor pathway inhibitor)	113	78
Radium-223	4	3
Radiation therapy for bone	6	4

**Table 2:** Efficacy results of ^177^Lu-iPSMA treatment in patients with mCRPC.

Overall Survival (OS)	^177^Lu-iPSMA	Control
	*n* = 52	*n* = 31
Deaths, *n* (%)	41 (79%)	27 (87%)
Censored cases	11 (21%)	4 (13%)
Median (days) (Kaplan–Meier estimate)	660	359
Ratio (95% CI of reciprocal ratio) (Kaplan–Meier estimate)	0.5439 (0.3405 to 0.8690)	
Hazard ratio log rank (95% CI)	0.5650 (0.3391 to 0.9412)	
*p*-value (log-rank test two-sided *p*-value)	<0.01	
**Overall Response Rate (ORR)**	**^177^Lu-iPSMA**	
Patients with evaluable disease (without censored cases)	*n* = 41	-
Complete response (CR), *n* (%)	4 (9.8%)	-
Partial response (PR), *n* (%)	11(26.8%)	-
ORR (CR + PR), *n* (%)	15 (36.6%)	-
**Biochemical and radiological molecular imaging response**	**Before** **treatment**	**After** **treatment**
Mean serum PSA levels (ng/mL) (range)	91 (41–217)	22 (4–56)
SUVmax in soft tissue tumor lesions (range)	54 (33–101)	14 (1–21)

**Table 3:** Efficacy results of ^177^Lu-DOTATOC treatment in patients with NET.

Overall Survival (OS)	All GEP-NET	Other NET
	*n* = 53	*n* = 28
Deaths, *n* (%)	35 (66%)	19 (68%)
Censored cases	15 (28%)	9 (32%)
Patients still alive at the cut-off date, *n* (%)	3 (5.7%)	0
Median (days) (Kaplan–Meier estimate)	1345	431
Ratio (95% CI of reciprocal ratio) (Kaplan–Meier estimate)	0.3353 (0.1831 to 0.6140)	
Hazard ratio log rank (95% CI)	0.1742 (0.05854 to 0.5182)	
*p*-value (log-rank test two-sided *p*-value)	<0.0001	
**Overall Response Rate (ORR)**	**All** **GEP-NET**	**Other NET**
Patients with evaluable disease (without censored cases)	*n* = 38	*n* = 19
Complete response (CR), *n* (%)	2 (5%)	0
Partial response (PR), *n* (%)	18 (47%)	0
ORR (CR + PR), *n* (%)	20 (52%)	0
Stable disease (SD), *n* (%)	17 (45%)	13 (71%)
Progressive disease	1 (3%)	3 (29%)
Disease control	97%	71%

## Data Availability

Not applicable.
